# Experiments upon Digestion

**Published:** 1856-09

**Authors:** Francis G. Smith

**Affiliations:** Professor of the Institutes of Medicine in the Medical Department of Pennsylvania College


### MEDICAL EXAMINER.

NEW SERIES.—NO. XLI.—SEPTEMBER, 1856.

### ORIGINAL COMMUNICATIONS.

### EXPERIMENTS UPON DIGESTION.

By Francis G. Smith, M. D.,

Professor of the Institutes of Medicine in the Medical Department of Pennsylvania College.

(Continued from July Number.)

The next question to be decided was, in regard to the influence of the gastric juice upon various alimentary substances, to wit: *albuminous, amylaceous, and oleaginous.*

It is very commonly accepted among physiologists, that the main business of the gastric juice is to act as the solvent of the albuminous, or nitrogenised articles of food, all the other alimentary principles either undergoing no change, or at farthest only a mechanical sub-division. It was reserved for Mialhe to show, that whatever albuminous aliment was presented to the solvent, it not only underwent solution, but was converted into a thoroughly new substance, a low form of albumen, and named by him *albuminose*, or, as Lehmann has proposed to call it, Peptone.* The formation of peptone depends solely on the action of the gastric juice, and occurs without the evolution or absorption of any gas, and without the production of any secondary substance.

Whatever article may have been selected for the production of peptone, the following properties may always be recognised. When reduced to the solid form by careful evapora-

* Phys. Chem. vol. ii. p. 50.

tion, peptone is a white, or yellowish-white substance; almost tasteless and inodorous; very soluble in water; but insoluble in alcohol of 83 per cent. Its watery solution reddens litmus, and is precipitated by chlorine, tannic acid and metallic salts, but is unaffected by boiling, by acids, or by alkalies. No precipitation or turbidity is produced by the addition of mineral or organic acids, either in a concentrated or in a very dilute state; even chromic acid fails to produce any appreciable effect.*

The *albuminose*, or *peptone*, thus formed by the action of the gastric juice, is not only soluble independently of the gastric juice, but is also capable of absorption, a circumstance which does not obtain in regard to albuminous substances generally, which penetrate animal membranes with difficulty, their diffusion equivalent, according to Graham, being exceedingly low, sugar being 8½ times and kitchen salt 19 times greater than albumen. On this lies the ground for the observation, that protein compounds, apparently all ready for use as nutriment, require a change by the digestive fluids previous to absorption. Without the gastric and intestinal juices, even soluble albumen and casein are taken up in far too little quantities to suffice for the nourishment of the frame.†

*Ex.* 10. As the representative of the *albuminous* class, four ounces of rarely done beefsteak were given to St. Martin at 10 A. M., May 5th, after a light breakfast of bread and coffee at 6 A. M., of the same day. No fluid was allowed to be taken in connection with the beef, nor any other article of food.

At 12 M., of same day, St. Martin was subjected to examination. On pushing back the fold of mucous membrane which acts as a valve to the fistulous orifice, a considerable amount of fluid was readily distinguishable in the stomach, mixed with bubbles of air; but no solid matter was visible. About a fluidounce and a half of this fluid was withdrawn from the stomach by a catheter, with the effect of producing nausea which precluded the possibility of obtaining more.

The fluid presented the same reaction as all the others experimented upon; s. g. 1009. Numerous flocculi were visible to the

* Op. cit.

† Lehmann, quoted by Chambers, "Digestion and its Derangement."

naked eye, looking like the debris of food and mucus. It was almost entirely inodorous, viscid, and to the taste decidedly acid.

The microscope, as in the other cases, revealed amorphous granular matter, mucous corpuscles, granular cells, and a few epithelial cells; a few transversely striated muscular fibres were also beautifully displayed, some almost uninjured, some broken down and with the sarcous elements liberated. Numerous oil globules were also distinctly visible, and a few fibres of yellow elastic tissue. The bulk of the material consumed as food had undergone entire solution, and had wholly lost its characteristic appearance.

A portion of the supernatant fluid was boiled actively, without, however, presenting the slightest trace of coagulation. The mineral acids had no effect upon it while cold, but when boiled with strong hydrochloric acid, the purple color of the protein bodies was distinctly manifested. The addition of acetic acid rendered the fluid rather more clear than before. The action of alkalies upon the fluid was not tried; but Trommer's test gave no evidence of the presence of glucose.

A portion of the dissolved material had doubtless been absorbed, but the quantity that still remained, over and above that withdrawn, was not the least remarkable circumstance in connection with the observation. All recent observers agree in stating, that the quantity of gastric fluid poured into the stomach is many times greater than that of the substance to be dissolved; this fluid, however, it must be remembered, is rapidly absorbed again, taking into the blood with it those alimentary matters which it has just dissolved and converted into peptone.

The conclusion from this observation is, that *the gastric juice is a true solvent for animal food.*

The *oleaginous* class of aliments was but imperfectly represented by the fat more or less commingled with the muscular fibres of the food administered as above. The number of oil globules visible under the microscope seems to confirm the observation of Bernard, that fatty matters undergo no change in the stomach beyond that of disaggregation.

Opinions are more divided as regards the influence of the gastric juice upon the *amylaceous*, or starchy articles of food. It is stated by Mialhe,* that this class of alimentary principles is converted into glucose by the digestive process, and that the saliva is the agent concerned, the conversion being continued even after the starch has descended into the stomach. In this opinion he is sustained by Lehmann, who asserts that grape sugar may be detected in the stomach in fifteen minutes after swallowing balls of starch, or after their introduction through fistulous orifices.† Carpenter also states, that the conversion of starch into sugar may go on in the stomach, but he quotes Frerichs as authority for the belief that it depends on the action of the saliva swallowed, the change not taking place when the œsophagus was tied so as to prevent the deglutition of that secretion, (*Human Physiology.*)

Bernard, on the other hand, denies that any such conversion takes place in the stomach, asserting that the acidity of the gastric juice arrests it, if it have already commenced in the mouth during mastication, or prevents it if it be introduced through a fistulous orifice.‡ Dalton holds the same opinion, which he maintains by experiments with gastric juice obtained from dogs, and by the introduction of starch into the stomachs of these animals through fistulous orifices.§ Both these latter observers, however, state that starch when acted upon by saliva out of the body, is converted into glucose, provided the saliva retain its alkalinity, but that the admixture of gastric juice arrests or prevents it if mixed with it. The following experiments may throw some light upon the subject, the amylaceous materials being represented by wheaten bread.

*Ex.* 11. On the sixth of May, a portion of wheaten bread was given to St. Martin while fasting, which he masticated deliberately and swallowed. In two hours and a half afterwards a portion of the contents of the stomach was removed for examination. The rèaction of this fluid was acid, and the microscopic appearances have been detailed already. (*Ex.* 1*st.*) Suffice it to say, that in addition to epithelial and mucous cells, starch granules, some whole, some broken, were distinctly recognisable.

* Mémoire sur la Digestion, &c.

† Dalton on Gastric Juice, in Amer. Journ. Med. Sci., Oct., 1854.

‡ MS. Notes of Bernard's Lectures, also Donaldson on Bernard's recent discoveries, Am. Jour. Med. Sc., Oct., 1851.

§ Am. Jour. Med. Sc. Oct., 1854.

After allowing the fluid to stand until it had settled, a portion of the supernatant liquid was tested with iodide of potassium and nitric acid, with the effect of manifesting decided evidence of the presence of starch by the production of the characteristic blue color. The same reaction was produced with the tinct. of iodine.

Another portion of the same fluid was subjected to Trommer's test; (solution of sulphate of copper and liquor potassæ;) the result showed the brick-dust red precipitate from the reduction of the oxide of copper, in very considerable quantity.

It may be objected to the above experiments, that dextrine or other organic matters of the fluid were instrumental in producing the reduction of the oxide of copper. ("Cette reaction est une oxydation égalément commune au sucre, á la dextrine, á la gomme, et á l'alcool. Il est utile d'ajouter encore que l'acide urique, l'urée, et l'albumine peuvent reduire ce reactif.")*

To meet this objection the fluid of digestion of bread was carefully filtered through animal charcoal, by which, as Bernard has shown, all matters, except glucose, are detained. The filtrate when subjected to Trommer's test, again afforded the brick-dust precipitate in large quantities.

*Ex.* 12. In order to ascertain, if possible, what effect the saliva might have had in producing the glycogenic change in the bread masticated, a portion of bread moistened with water was introduced through the fistulous orifice, and St. Martin was requested to swallow as little saliva as possible, which, as he used tobacco, he had little difficulty in complying with. In an hour and a half afterwards, the contents of the stomach were withdrawn. The same acid reaction was manifest, the same microscopic appearances, and the same solution of the materials were present, although not to the same degree as when the bread was masticated.

The fluid was carried through the same test with a like result, viz: faint evidences of starch and decided evidences of glucose.

Those who have read the admirable paper of Dr. J. C. Dalton, on "Gastric Juice and its Office in Digestion,"† will remember that he states, that "the presence of gastric juice interferes with Trommer's test for grape sugar," and further, that in animals

* Bernard, Leçons de Physiologie Experimentale Appliquée a la Medicine, 1855.

† Amer. Jour. Med. Sc, Oct., 1854.

fed with starch, "no sugar is to be detected at any time" in the product of digestion, and still further, that if gastric juice be added to a mixture of saliva and starch, "there is no trace of sugar, even at the end of three hours, and the starch retains its usual properties." Dr. Dalton's experiments were performed upon dogs, in whom he had established fistulous communications with the stomach. It is possible that the greater acidity of their gastric juice may have prevented the metamorphic change, as the writer has witnessed in saliva acidulated with hydrochloric acid; but in the human subject, if we are to accept St. Martin as evidence, his observations have not been verified, indeed they have been disproved.*

The *conclusions* from the foregoing experiments are, that starchy materials are digested in the human stomach; that human gastric juice does not prevent the conversion of starch into grape sugar; and that this conversion may take place in the stomach, independently of the action of saliva, for, as Bernard has shown, any mucous membrane and some alkaline fluids, as the serum of the blood, possess the same power.

In presenting these experiments to those interested in such matters, it is not claimed for them that they are decisive; they are frankly offered as a contribution towards settling a "quæstio vexata." Of their value, others must judge. Their prosecution was a subject of deep interest to the writer, whose chiefest regret in relation to them is, that the opportunity was too fleeting to enable him to obtain more decided and extensive results.

---

*Cases of Intermittent Fever.* By W. A. PECK, M. D., Berwick, Pennsylvania.

CASE I.† Mary W——, a hale German girl, of 18 years, on the 25th of August, 1855, had an attack of uncomplicated remittent fever, which readily yielded to the usual remedies. This fever was succeeded, after a week's convalescence, by paroxysms of a retarding quotidian intermittent. These she continued to

*It may be mentioned in passing, that cane sugar was also converted into grape sugar in St. Martin's stomach. In the product of digestion of calves' feet jelly, glucose was distinctly recognisable. The gelatine was unchanged.

